# Reference dosimetry data and machine performance comparison for tomotherapy delivery systems audited by IROC

**DOI:** 10.1002/acm2.70390

**Published:** 2025-11-18

**Authors:** Paige A. Taylor, Hunter Mehrens, Mallory C. Glenn, Fre'Etta Brooks, Hayden Scott, Lian Duan, Taylor Meyers, Diana Carrasco Rojas, Michael Yang, Paola Alvarez, Jessica Lowenstein, Andrea Molineu, Stephen F. Kry

**Affiliations:** ^1^ Department of Radiation Physics The University of MD Anderson Cancer Center Houston Texas USA; ^2^ Medical Physics Graduate Program The University of Texas MD Anderson Cancer Center Graduate School of Biomedical Sciences Houston Texas USA; ^3^ University of Washington Department of Radiation Oncology Seattle Washington USA

**Keywords:** audit, quality assurance, TomoTherapy

## Abstract

The aim of this work was to compare the imaging and radiation oncology core (IROC) on‐site TomoTherapy dosimetry measurement results among institutions and generate a dataset of basic dosimetric properties for TomoTherapy units. Independent ion chamber measurements for nine TomoTherapy units were acquired by IROC physicists between 2008 and 2023. Measurements included percent depth dose (PDD) in a water tank and off axis factors (OAF) in a solid Polystyrene phantom. The independent measurements collected during the on‐site audits for each TomoTherapy system were compared to corresponding TomoTherapy TPS calculations from the institution to assess agreement. Numeric measurement values and comparative ratios are compiled and tabulated according to measurement type (i.e., PDD and OAF). The distribution of the measurements was evaluated, and the mean, standard deviation of the mean, and median values are reported. These data provide an independent guide describing the inherent performance characteristics of TomoTherapy units. Such data may serve as a secondary check for users wishing to verify their beam model commissioning and ongoing quality assurance efforts.

## 1 INTRODUCTION

The TomoTherapy (Accuray, Inc., Sunnyvale, CA; TomoTherapy Inc. prior to 2011) delivery system^1^ is a dedicated device that delivers helical intensity modulated radiation therapy (IMRT) with integrated image guidance using megavoltage computed tomography (MVCT).^2^ These unique systems follow a standard beam modeling approach; that is, TomoTherapy systems are initially tuned so the linac performance matches standard beam data.^3^, ^4^ This approach ideally limits the need for user‐generated modeling input and minimizes variability among machines.^5^ Ultimately, the user must validate that the machine performance matches the treatment planning system (TPS) model,^4^, ^6^, ^7^, ^8^, ^9^ often with little external guidance or support.

To support high quality radiation therapy, the American Association of Physicists in Medicine (AAPM) recommends external reviews as a part of linac commissioning and ongoing quality assurance (QA).^10^ The Imaging and Radiation Oncology Core (IROC) provides on‐site audits as one such method of validation. During an on‐site audit, an IROC physicist performs independent dosimetry measurements on the institution's linear accelerators. These measurements are compared with the institution's TPS to evaluate how well the institution has modeled the linac's performance and verify that appropriate radiation doses are delivered in the clinic.

From these independent measurements, IROC has been able to publish datasets describing community linac performance for both Varian and Elekta machines.^11^, ^12^ These references now serve as guidance for clinical use for physicists to compare their beam parameter value to others in the community. Leveraging these datasets allows for a more cohesive and accessible way to help clinics improve their treatment accuracy.

There is a great need to expand upon the limited work that has compared the general dosimetric characteristics among TomoTherapy units.^2^ The goal of this work is to compare basic machine performance characteristics of various TomoTherapy units, determine the current state of implementation among institutions, and provide a standard dataset for community comparison. Using this information, those commissioning helical‐type delivery systems may have a point of reference with which to compare their measurements and TPS modeling.

## 2 MATERIALS AND METHODS

### 2.1 Data collection

The current study uses data from nine different TomoTherapy treatment machines (Hi‐Art and TomoHDA) at seven institutions audited between 2008 to 2023. Independent measurements were used for comparison against the institutions’ TPS calculation values under the same conditions. During an on‐site audit, IROC physicists visit an institution and collect point dose measurements using standard geometric conditions, similar to a periodic machine quality assurance (QA) check. Institutions are asked to provide TPS calculated values using the same geometric conditions for a direct comparison between the on‐site measurements and the institution's TPS calculation. Site visit measurements are evaluated based on IROC's established internal protocol; the agreement criterion for relative measurements is ± 2%.

The percentage depth dose (PDD) was measured during IROC site visits using a 30 × 30 × 30 cm^3^ water tank (provided by IROC) placed at 85 cm source‐to‐surface distance. Measurements were made with an IROC Exradin A12 chamber (Standard Imaging, Madison, WI, USA) calibrated at an accredited dosimetry calibration laboratory (ADCL). The PDD was measured in a 5 × 40 cm^2^ and a 2.5 × 40 cm^2^ field at effective depths of 5, 10, and 15 cm. The 1 × 40 cm^2^ field size was not measured due to the size limitations of the reference chamber. The beam was delivered in static (not helical) mode, with the couch also static. Measurement time was 1–1.5 h, with additional time for water tank setup and alignment.

The off‐axis factors (OAF) were obtained using an IROC PTW TN30013 Farmer‐type chamber (PTW, Freiburg, Germany) inserted into a solid polystyrene phantom placed on top of the institution's solid water phantom. The phantom was designed for fixed positions at each off‐axis point of measurement. The OAFs were measured at an effective point of measurement of 1.5 cm at distances of 5, 10, and 14 cm to the left and right of the central axis (CAX) in a 5 × 40 cm^2^ field. This field size was chosen as a sampling point of overall model performance. The beam was delivered in static (not helical) mode and the couch was static. Measurement time was 1–1.5 h, including setup.

All measurements were collected at the effective point of measurement of the ion chamber; 0.6*r*_cav_ upstream of the physical center of the chamber. Each measurement was performed twice, averaged, and immediately compared to the TPS calculation. If the comparison between the on‐site measurement and the institution's TPS exceeded IROC's ± 2% criterion, setup geometry was confirmed, and then two additional measurements were performed. If a discrepancy persisted, the results were compared to the institution's most recent annual QA. All data were reviewed by a second IROC physicist to limit potential transcription errors and confirm calculational accuracy.

### 2.2 Data analysis

Mean, standard deviation of the mean, and median values were recorded for both independently acquired IROC measurements and institutional TPS calculated dosimetry data. A ratio between the measured data and the institutional TPS calculation at a given point was calculated to assess the agreement between the two (defined as IROC measurement/institutional TPS calculation). This was done for each field size and depth for PDD measurements, and each off‐axis distance for both the right and the left off‐axis factors. Linear regression was used to analyze changes in TomoTherapy audits over time.

For the OAFs, the Wilcoxon rank‐sum test was used to measure the statistical significance between the left and right off‐axis factors. Mean values for the given parameters were tested to determine the presence of any systematic bias between the measured and calculated values. It was determined that any significant statistical (*p* ≤0.05) or clinical differences (values exceeding IROC's ± 2% site visit criteria) in the distribution represented machine parameters that did not match manufacturer data. IBM SPSS 24 (Armonk, NY) was used to perform all statistical tests and linear regression.

## 3 RESULTS

### 3.1 Percent depth dose

**FIGURE 1:**
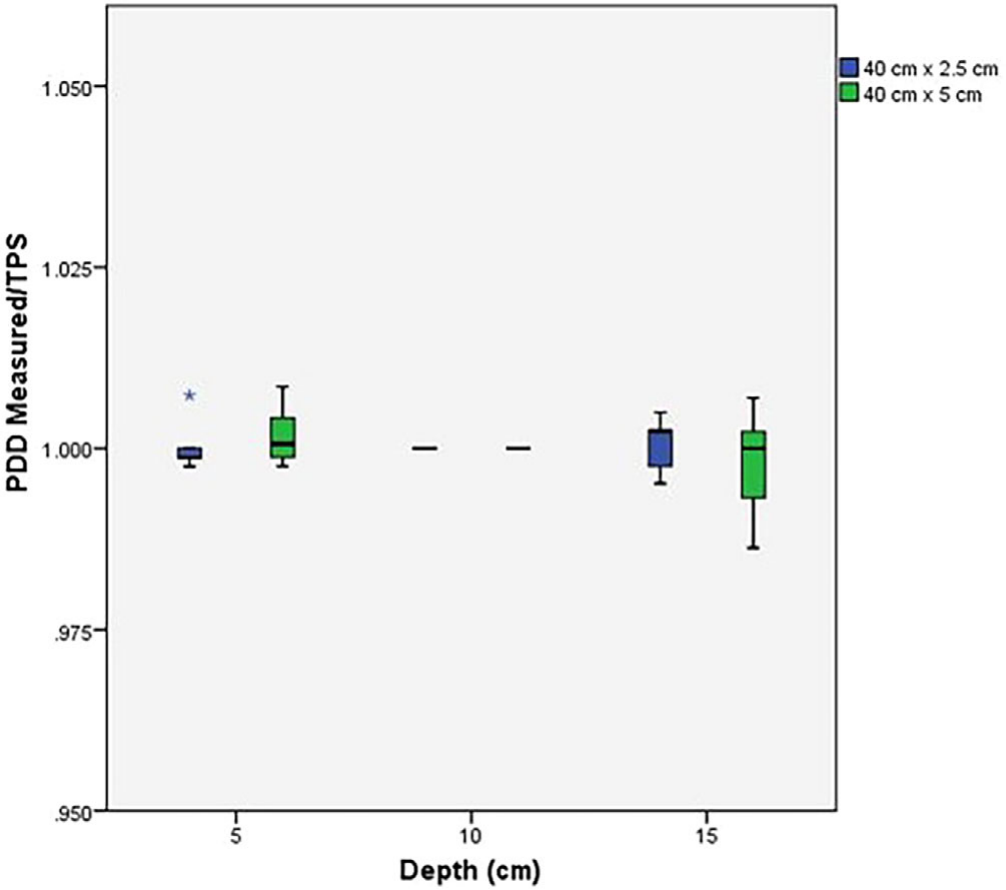
Box plot of IROC‐measured/TPS percent depth doses (PDDs) for 40 cm × 2.5 cm (blue) and 40 cm × 5 cm (green) field sizes at depths of 5, 10, and 15 cm. Reference is taken at 10 cm depth for both field sizes. The median ratio for 5 and 15 cm is close to 1; however, the spread of the data increases with depth.

The data for the percent depth dose measurements for the TomoTherapy machines is reported in Table 1. Figure 1 provides a boxplot of both PDD measurements for measured/TPS by depth. The IROC measured and TPS values for the percent depth dose measurements showed good agreement. All Measured/TPS ratios were within 1.4% for the 5 cm × 40 cm field size, and within 0.7% for the 2.5 cm × 40 cm field size. The full data set can be found in the Appendix in tables A1 and A2.

**TABLE 1:** Mean and median values for the IROC measured, TPS, and Measured/TPS data.

		IROC Measured		Institution TPS		Measured/TPS	
	Depth (cm)	Mean ± St Dev	Median	Mean ± St Dev	Median	Mean	Median
PDD 5 cm × 40 cm	5	82.2 ± 1.2	81.8	82.1 ± 0.9	81.9	1.00	1.00
	10^†^	60.3 ± 0.9	60.0	60.3 ± 0.9	60.0	1.00	1.00
	15	43.7 ± 0.6	43.6	43.8 ± 0.7	43.7	1.00	1.00
PDD 2.5 cm × 40 cm	5	80.3 ± 1.4	79.7	80.3 ± 1.1	79.8	1.00	1.00
	10^†^	57.4 ± 1.0	57.0	57.4 ± 1.0	57.0	1.00	1.00
	15	40.9 ± 0.7	40.6	40.9 ± 0.7	40.7	1.00	1.00

†Values normalized to 10 cm depth.

Abbreviation: PDD, percent depth dose.

### 3.2 Off‐axis factors

**FIGURE 2:**
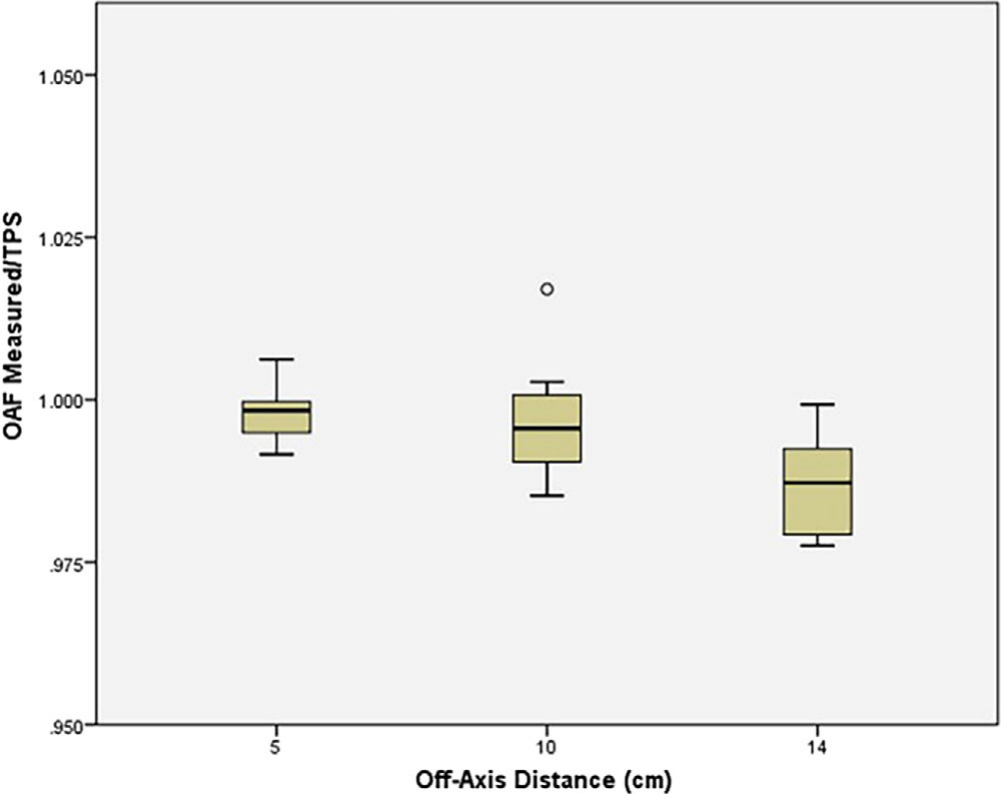
Box plot of IROC‐measured/TPS average off‐axis factor (OAF) by depth. As off‐axis distance increases, median ratio decreases away from 1 and the range of values increases. For 10 cm, one outlier occurs which is 2% high.

The data for the off‐axis factors (OAFs) are shown in Table 2. Although measurements were taken for OAFs in both the left and right directions, no statistical significance was identified between the two sides for the measured data (Wilcoxin rank‐sum test, *p* > 0.05), so the data were averaged. Figure 2 provides a boxplot of the average OAF for measured/TPS by distance off axis. Two OAF ratios were outside of IROC's ± 2% criterion, with one data point disagreeing by 4.5%. This suggests poor modeling of the beam profile relative to the standard manufacturer machine data. The full data set can be found in the Appendix.

**TABLE 2:** Off‐axis factor (OAF) mean and median values for the IROC measured, TPS, and Measured/TPS data. The range of Measured/TPS ratios is also shown, with results outside of ± 2% representing measurements failing IROC's criterion. Measured/TPS median calculated as the median of all ratios.

		IROC measured		Institution TPS		Measured/TPS		
	Off‐axis distance(cm)	Mean ± St Dev	Median	Mean ± St Dev	Median	Mean ± St Dev	Median	Range
OAF 5 cm × 40 cm	5	0.892 ± 0.008	0.893	0.894 ± 0.005	0.896	1.00 ± 0.01	1.00	[0.98 ‐ 1.01]
	10	0.734 ± 0.008	0.733	0.736 ± 0.006	0.738	1.00 ± 0.01	1.00	[0.98 ‐ 1.02]
	14	0.624 ± 0.009	0.625	0.632 ± 0.006	0.631	0.99 ± 0.01	0.99	[0.96 ‐ 1.01]

### 3.3 Machine performance over time

From 2008–2023, neither the percent depths doses nor the off‐axis factors showed a statistically significant difference over time (linear regression, *p* > 0.05). Although repeat measurements of the same machine (at separate time points) are not included in these datasets, reproducibility of IROC measurements was assessed between repeat audits of the same machine. The results demonstrate consistency across the machines; repeat measurements were within 1.1%, and on average differed by 0.4%

## 4 DISCUSSION

Our current study evaluated point dose measurements for PDD and OAFs to assess agreement between independent measurements conducted during IROC on‐site audits and corresponding TPS calculations from the institution. For the ratio of IROC measured vs. TPS, OAFs demonstrated more discrepancies when compared to PDDs. Several OAF ratios were outside of IROC's ± 2% criterion, with one data point disagreeing by 4.5%, suggesting that the standard beam model does not match the physical beam profile well. The largest discrepancies were observed in both directions at the greatest distance off‐axis (14 cm). The change in TomoTherapy audit results over time was evaluated using linear regression, but no trend was observed.

Similar trends in TomoTherapy were observed in the Varian and Elekta standard data sets previously reported by IROC; for example, minor differences were observed between measured and planned PDDs for Varian and Elekta machines.^11^, ^12^ These differences were typically less than one standard deviation. Furthermore, larger differences were observed between measured and planned OAFs across all machines; this depended on the linac type for Varian machines, with Measured/TPS ratios exceeding one standard deviation for many C‐series linacs.^11^, ^12^ One difference between the TomoTherapy data compared to the Varian and Elekta data is that a single TPS was used for the TomoTherapy machines in this data set, and that TPS differs from those used by Varian and Elekta. Ideally the TomoTherapy TPS is well‐modeled for the TomoTherapy machine, but there is not a secondary point of comparison for our analysis. RayStation TPS (RaySearch Laboratories, Stockholm, Sweden) now can be used for TomoTherapy machines. Although none of the data in this study used the RayStation TPS, it could potentially provide a useful validation of results. Although the institutional datasets from their TPS assumed symmetry between the right and left off‐axis factors, it's important for users to verify whether the beam is symmetric during commissioning.

This study evaluated nine units between 2008 and 2023. The data did not show a trend with time, suggesting newer machines in the analyzed cohort were not significantly different from older machines. IROC historically has used a minimum of five data sets to calculate reference data, which provides sufficient data to draw conclusions, especially given TomoTherapy's unique approach to modeling, where TomoTherapy machines are tuned so the linac performance matches standard beam model data, instead of the other way around. Currently there are several TomoTherapy treatment machine models: Hi‐Art, TomoHDA, and Radixact. Most of the data presented in this paper is from the Hi‐Art machine model, while no data points were measured for the Radixact machine. Although each model has upgraded features that align with new scientific advancements, the fundamental dose delivery process is the same. A treatment planning study by Kurosaki et al. showed no significant differences in dose distributions for planning target volumes between Hi‐ART, Tomo‐HD, and Radixact.^13^ It may be feasible to apply these results to all three treatment machine models, but it would be useful to obtain Radixact machine measurements to validate.

## 5 CONCLUSION

This work compiles measurements from nine TomoTherapy machines to generate a reference dataset describing their basic dosimetric performance, specifically for PDD and OAF values. The dataset produced in this research serves as a secondary verification tool for an institution's measurements, which may aid in the identification of anomalies that require further attention. Any substantial disparities should be investigated and verified.

## AUTHOR CONTRIBUTIONS

Paige A. Taylor: Substantial contributions to the conception of the work, analysis and interpretation of data, drafting, and final approval of the version to be published; Hunter Mehrens: Substantial contributions to the conception of the work, analysis and interpretation of data, drafting, and final approval of the version to be published; Mallory C. Glenn: analysis and interpretation of data, drafting, and final approval of the version to be published; Fre'Etta Brooks: Interpretation of data, drafting, and final approval of the version to be published; Hayden Scott: interpretation of data, drafting, and final approval of the version to be published; Lian Duan: Interpretation of data, drafting, and final approval of the version to be published; Taylor Meyers: interpretation of data, drafting, and final approval of the version to be published; Diana Carrasco Rojas: Interpretation of data, drafting, and final approval of the version to be published; Michael Yang: Interpretation of data, drafting, and final approval of the version to be published; Paola Alvarez: Substantial contributions to the conception of the work, interpretation of data, drafting the work, and final approval of the version to be published; Jessica Lowenstein: acquisition of data, revising the work critically for important intellectual content, and final approval of the version to be published; Andrea Molineu: acquisition of data, revising the work critically for important intellectual content, and final approval of the version to be published; Stephen F. Kry: Substantial contributions to the conception or design of the work, analysis and interpretation of data, drafting, and final approval of the version to be published.

## CONFLICT OF INTEREST STATEMENT

The authors declare no conflicts of interest.

## Appendix

See Tables A1 and A2.

**TABLE A1:** Percent depth dose for 40 cm × 5 and 40 cm × 2.5 cm field sizes.

	PDD for 40 cm × 5 cm			PDD for 40 cm × 2.5 cm		
	Depth, cm			Depth, cm		
Machine	5	10	15	5	10	15
Machine 1, Hi‐Art						
IROC^a^	81.7	60.1	43.6	79.5	56.9	40.6
Institution^b^	81.9	60.1	43.6	79.7	56.9	40.5
Machine 2, Hi‐Art						
IROC	82.9	60.6	43.8	79.7	57.0	40.6
Institution	82.2	60.6	44.2	79.8	57.0	40.7
Machine 3, Hi‐Art						
IROC	81.6	59.7	43.3	79.2	56.5	40.3
Institution	81.6	59.7	43.2	79.3	56.5	40.1
Machine 4, Hi‐Art						
IROC	81.9	59.9	43.5	N/A		
Institution	81.8	59.9	43.4			
Machine 5, Hi‐Art						
IROC	81.2	59.5	43.2	N/A		
Institution	81.2	59.5	43.8			
Machine 6, Hi‐Art						
IROC	N/A			N/A		
Institution						
Machine 7, Hi‐Art						
IROC	81.0	59.4	43.2	N/A		
Institution	81.2	59.4	42.9			
Machine 8, TomoHDA						
IROC	82.7	60.8	44	80.6	57.5	40.9
Institution	82.6	60.8	44.2	80.6	57.5	41.1
Machine 9, Hi‐Art						
IROC	84.6	62.0	45.0	82.6	59.0	42.1
Institution	84.0	62.0	45.0	82.0	59.0	42.0

^a^ Independent dosimetry measurements on the linear accelerator by an Imaging and Radiation Oncology Core physicist.

^b^ Calculation from the institution's treatment planning system.

Abbreviation: PDD, percent depth dose.

**TABLE A2:** Off‐axis factors for 40 cm × 5 cm field size.

	OAF, Left of CAX			OAF, Right of CAX		
	Distance from CAX, cm			Distance from CAX, cm		
Machine	5	10	14	5	10	14
Machine 1, Hi‐Art						
IROC^a^	0.892	0.730	0.617	0.893	0.730	0.621
Institution^b^	0.895	0.735	0.629	0.891	0.734	0.627
Machine 2, Hi‐Art						
IROC	0.890	0.729	0.621	0.884	0.727	0.622
Institution	0.899	0.739	0.631	0.890	0.731	0.625
Machine 3, Hi‐Art						
IROC	0.881	0.739	0.628	0.903	0.754	0.636
Institution	0.888	0.730	0.635	0.896	0.738	0.630
Machine 4, Hi‐Art						
IROC	0.904	0.740	0.632	0.870	0.715	0.596
Institution	0.900	0.740	0.632	0.886	0.729	0.624
Machine 5, Hi‐Art						
IROC	0.892	0.735	0.627	0.892	0.731	0.616
Institution	0.896	0.738	0.631	0.891	0.732	0.626
Machine 6, Hi‐Art						
IROC	0.902	0.739	0.628	0.893	0.730	0.621
Institution	0.899	0.739	0.645	0.885	0.726	0.631
Machine 7, Hi‐Art						
IROC	0.894	0.733	N/A	0.889	0.727	N/A
Institution	N/A			N/A		
Machine 8, TomoHDA						
IROC	0.896	0.739	0.633	0.893	0.737	0.631
Institution	0.896	0.739	0.635	0.896	0.739	0.635
Machine 9, Hi‐Art						
IROC	0.900	0.737	0.626	0.894	0.733	0.624
Institution	0.900	0.746	0.638	0.900	0.746	0.638

^a^ Independent dosimetry measurements on the linear accelerator by an Imaging and Radiation Oncology Core physicist.

^b^ Calculation from the institution's treatment planning system.

Abbreviations: CAX, central axis; OAF, off‐axis factor.
